# Relationship Between Second Victim Experience and Turnover Intention in Chinese Nurses: Assessing the Mediating Role of Posttraumatic Growth Using Structural Equation Modeling

**DOI:** 10.1155/jonm/7327139

**Published:** 2025-06-26

**Authors:** Yun Xu, Qi Jin, Qinghua Zhou, Rong Zhang, Weiyan Ding

**Affiliations:** ^1^Department of Nursing, The First Affiliated Hospital, Zhejiang University School of Medicine, Hangzhou, China; ^2^Chengde Medical University, Chengde, China

**Keywords:** adverse event, posttraumatic growth, resilience, second victim, turnover intention

## Abstract

**Aim:** This study aimed to investigate the mediating effect of posttraumatic growth in the relationship between second victim experience and turnover intention, as well as the moderating effect of resilience among nurses who have experienced second victimization.

**Background:** Healthcare professionals, particularly nurses, are vulnerable to becoming “second victims” following adverse events. However, the prevalence of second victim experiences among nurses has been largely overlooked, and limited attention has been given to the relationship among posttraumatic growth and turnover intention.

**Methods:** A cross-sectional survey was conducted using convenience sampling. A total of 572 nurses participated in the study, with data collected via the Questionnaire Star Platform. The study utilized a general information questionnaire, the Resilience Scale, the Posttraumatic Growth Inventory, the Second Victim Experience and Support Tool, and the Turnover Intention Scale to assess relevant variables.

**Results:** The average scores of second victim experience, resilience, posttraumatic growth, and turnover intention were (64.86 ± 11.32), (26.52 ± 8.13), (62.12 ± 11.32), and (16.05 ± 4.42), respectively. The second victim experience was positively correlated with turnover intention (*r* = 0.372, *p* < 0.001), and the posttraumatic growth partially mediated the relationship between second victim experience and turnover intention, with an indirect effect of −0.015 (95% CI = −0.0027∼−0.006). The relationships between second victim experience and turnover intention, as well as the mediating effect of posttraumatic growth, were moderated by resilience (*p* < 0.05).

**Conclusion:** The second victim experience among nurses following adverse event has a significant mediating effect on turnover intention. Additionally, resilience moderates both the direct and indirect pathways in the model linking second victim experience, posttraumatic growth, and turnover intention.

**Implications for Nursing Management:** The second victim phenomenon presents a significant challenge and warrants greater attention. Hospital managers should recognize the impact of second victim experiences, foster a supportive and safe practice environment, and provide psychological support to protect healthcare professionals' mental health, reduce turnover intention, and enhance nursing quality and safety.

## 1. Introduction

Patient safety is still the core of medical services and a serious global public health concern. The World Health Organization (WHO) estimates that, in 2022, one in 10 patients worldwide will experience an adverse event. In 2000, Wu first introduced the concept of the “second victim” (SV) [1], and Scott et al. defined SVs as “healthcare providers who experience physical and psychological trauma due to unanticipated medical errors or patient-related injuries” [2]. As primary caregivers and at the forefront of patient care, nurses are particularly vulnerable to becoming SVs [3]. They might be the SVs once or multiple times, leading to varying degrees and forms of psychosomatic trauma, such as personal distress, insomnia, burnout, guilt and self-doubt, or professional identity dissipation, which this condition is referred to as SV syndrome [4–10]. SV syndrome has been shown to have long-term adverse effects on both healthcare professionals and patients. While most healthcare professionals may recover naturally, the traumatic experience can profoundly impact their personal and professional lives, potentially compromising job performance and the ability to deliver safe patient care. As essential contributors to patient safety, healthcare professionals play a critical role in maintaining high-quality care standards. Recognizing this, the WHO's World Patient Safety Day campaign (2020-2021) emphasized the importance of healthcare worker safety as a fundamental priority for enhancing overall patient safety. The campaign also underscored the importance of reporting and analyzing serious incidents related to safety and the need to reduce work-related stress and burnout.

According to stress theory, individuals who experience trauma exhibit stress responses of varying intensity, depending on the severity of the trauma [11]. For nurses, adverse events often trigger complex responses, including feelings of guilt, fear, shame, self-blame, sleep disturbance, and other psychosomatic symptoms [12]. These responses can decrease their sense of job competence and achievement, potentially leading to doubts about their professional abilities and career development. Consequently, this may result in burnout, turnover intention, and even actual turnover [3, 13]. The SV phenomenon continues to affect medical staff negatively, and higher turnover intention increases the likelihood of further adverse events, creating a vicious cycle [14]. High nurse turnover rates adversely affect various stakeholders, including health systems, organizations, medical staff, and patients. Defensive behaviors may emerge among SVs, and if not addressed promptly, these behaviors can increase the likelihood of errors in daily nursing practice. A cross-sectional survey of 1163 nurses in Singapore found that approximately 31.8% of clinical nurses intended to leave their jobs after experiencing adverse events [15]. While previous studies have confirmed the relationship between SV experience and turnover intention, the underlying mechanisms remain unclear.

Scott et al. [2] noted that, in addition to leaving their current positions or workplaces, a potential outcome of experiencing an adverse event is learning from the experience and enhancing one's capabilities. This positive psychological change resulting from trauma is known as posttraumatic growth (PTG), which was first proposed by psychologists Tedeschi and Calhoun in the mid-1990s, and represents a form of affirmative psychological transformation [16]. Individuals who experience traumatic events may develop better levels of functioning. PTG is not an automatic result of trauma but arises from cognitive restructuring, which is a result of personal efforts to cope with or survive the traumatic experience; they are catalysts for the creation of meaning, a necessary step in transforming an old (negative) schema into a more positive view. This process positively changes individuals' perceptions of themselves and their work, influencing their professional attitudes and motivation [16]. However, the incidence of PTG in workplace trauma is generally lower compared to other life trauma events [17]. Nurses who experienced severe distress may struggle to find meaning or positive outcomes from traumatic events [17, 18]; a previous study found that nurses with higher levels of PTG were likely to develop turnover intention, but only when they also received adequate organizational support [3, 19]; when PTG occurs, it can mitigate the negative effects of SV experience by helping nurses reframe their experiences and find new meaning in their life. Other researchers showed that PTG can lead to positive and negative outcomes, including increased turnover intention, as nurses who experience growth may seek new challenges or career opportunities [20]. Consequently, we hypothesize that nurses' SV experience not only directly impact the turnover intention but may also exert an indirect influence through PTG.

Resilience, or psychological resilience, refers to an individual's capacity to adapt to challenges and recover from adversity [21]. As a crucial internal positive psychological resource, resilience helps individuals bounce back from negative states such as anxiety, sadness, and self-doubt after trauma. It can improve nurse's perceived professional benefits and job satisfaction and reduce job burnout and stress [22]. Previous research has suggested that resilience is a potential protective factor for PTG and positively correlates with PTG [23]. With higher levels of resilience, individuals tend to adopt a more positive and optimistic attitude toward traumatic events, leveraging personal and external resources to cope, enhancing their adaption and development. Some researchers believe that resilience can significantly predict PTG [24, 25], and individuals who experience PTG tend to have more positive attitudes toward their careers, showing greater willingness to remain in their current jobs [22].

**Figure 1:**
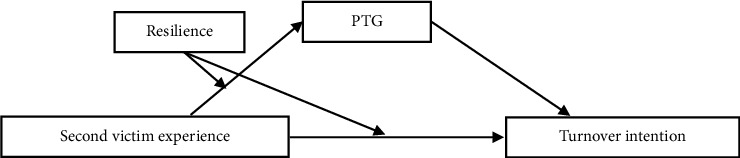
Hypothesis model: relationship between second victim experience and turnover intention.

The current preliminary study focused on nurses and aimed to explore the direct and indirect effects of SV experience on turnover intention, with PTG as a mediator. In addition, we examined the moderating role of resilience in shaping these relationships to provide context-specific insights into how SV experience, resilience, and PTG interact to influence turnover intention among Chinese nurses. We propose a moderated mediation model (Figure 1) to examine the relationships among nurses' SV experience, resilience, PTG, and turnover intention in China.

## 2. Methods

### 2.1. Study Design

This cross-sectional survey was conducted in Hangzhou, south of China, and was approved by the Ethics Committee of the First Affiliated Hospital of Zhejiang University, School of Medicine (approval number: 2024YD0823). Participation in the study was voluntary and participants provided written informed consent. Data from this study were kept strictly confidential. In order to guarantee a comprehensive and accurate research reporting, we used the Strengthening the Reporting of Observational Studies in Epidemiology (STROBE) guideline for cross-sectional studies, and all methods were performed in accordance with the relevant guidelines and regulations.

### 2.2. Data Procedure

The online survey data were collected from August 1^st^ to 15^th^ 2024, using the Questionnaire Star Software (Wenjuanxing) in the present study. Participants accessed the survey via a link or QR code, completing the questionnaire anonymously after informed consent. The survey was conducted in Mandarin, with each phone number restricted to a single response to prevent duplicate submissions. The survey included a screening question to identify nurses who had experienced adverse events (e.g., falls, medication errors, violent attacks, or other patient safety–related incidents). Nurses who responded “No” to this question were directed to the end of the survey and excluded from further analysis. Participants were recruited from several tertiary Grade-A hospitals in Hangzhou, Zhejiang Province, China. The inclusion criteria were (1) registered nurse, (2) at least 6 months of clinical experience (direct patient care), and (3) experienced adverse events. The exclusion criteria were (1) interns or trainee nurses and (2) nurses who declined to participate. A total of 1171 questionnaires were filled out, of which 560 (47.82%) respondents reported no experience with adverse events and were excluded. Among the remaining 611 responses, 572 questionnaires were deemed valid for analysis, resulting in an effective response rate of 93.62%. The survey was accessible via https://www.wjx.cn/vm/rLb5DR1.aspx.

### 2.3. Measures

The home-made survey comprised 5 sections: sociodemographic information (e.g., age, gender, marital status, department, and years of working), the Second Victim Experience and Support Tool (SVEST), the Chinese-Posttraumatic Growth Inventory (C-PTGI), the Resilience Scale, and the Turnover Intention Scale.

#### 2.3.1. SVEST

The Chinese version of SVEST, translated by Chen and colleagues [26], was used to assess the SV experience in this study. This 24-item questionnaire comprises six dimensions: psychological distress, physical distress, practice distress, colleague support, management support, and no work-related support. It is scored by a 5-point Likert scale with anchors ranging from 1 (completely disagree) to 5 (completely agree). The total score ranges from 24 to 120, with reverse-scored items (3 support dimensions) being converted, and higher scores indicate more severe the SV symptoms and less support received. The overall Cronbach's *α* of the Chinese version of the SVEST was 0.824, with Cronbach's *α* of each dimension ranging between 0.666 and 0.910. This study's overall Cronbach's α was 0.814, with each dimension ranging between 0.773 and 0.943.

#### 2.3.2. C-PTGI

The C-PTGI, revised by Wang et al. [27], was used to assess PTG. This inventory consists of 20 items and can be divided into 5 dimensions: relationship with others, new possibilities, personal strength, self-transformation, and appreciation of life. A 6-point Likert scale measures the responses ranging from 0 (not at all) to 5 (very much). The total scores range from 0 to 100, with higher scores indicating higher PTG levels. The overall Cronbach's *α* was 0.874, with dimension-specific Cronbach's *α* ranging from 0.611 to 0.796. The overall Cronbach's *α* was 0.970, with dimension-specific Cronbach's *α* ranging from 0.853 to 0.929 in this study.

#### 2.3.3. Resilience Scale

The resilience scale was developed by Connor and Davidson in 2003 [28]. The original scale consisted of 25 items, but a shorter version, such as the 10-item resilience scale, was validated for use in various populations. The Chinese version of the 10-item Resilience Scale, translated by Ye et al. [29], was used in this study. This unidimensional scale is scored by a 5-point Likert scale, ranging from 0 (never) to 4 (always), leading to a summed score ranging from 0 to 40, with a higher score indicating a higher level of resilience. Cronbach's *α* was 0.851, and the split-half reliability was 0.811. Cronbach's *α* was 0.921 in this study.

#### 2.3.4. Turnover Intention Scale

Michaels and Spector developed the turnover intention scale in 1982 [30], and the Chinese version of the scale was translated by Li and colleague [31]. The scale consists of 3 dimensions with a total of 6 items. The turnover intention I dimension was formed by items 1 and 6, which indicates the likelihood of quitting the current job; items 2 and 3 form the turnover intention II dimension, representing the motivation to seek another job; the last items 4 and 5 form turnover intention III, indicating the likelihood of obtaining an external job. A 4-point Likert scale is used to measure the items with reverse scoring. A higher summed score on the scale indicates a stronger turnover intention. The overall Cronbach's *α* for the scale was 0.773. Cronbach's *α* was 0.894 in this study.

### 2.4. Statistical Analyses

Data were analyzed using SPSS Version 26 and AMOS Version 26. The demographic characteristics of the participants as well as levels of the four major variables (SVE, PTG, resilience, and turnover intention) were measured using frequency, percentage, mean, and standard deviation. Correlations among the major variables were measured using Pearson's correlation coefficient. Mediation and moderation effects were tested using the Process macro for SPSS (Model 4 and Model 8) with a 5000 bootstrap sample. The bootstrapping method provides the most precise 95% confidence intervals and determines the significance levels for indirect effects.

## 3. Results

### 3.1. Demographic Characteristics of Samples

Among the 572 participants, 537 (93.89%) were female nurses, a total of 173 (30.24%) had heard “second victim” before, and most participants had completed a bachelor degree (*n* = 513, 89.69%) and 190 (33.22%) worked in Internal Medicine department, details referred to Table 1.

### 3.2. Correlations Among SV Experience, Resilience, PTG, and Turnover Intention of Nurses

**Figure 2:**
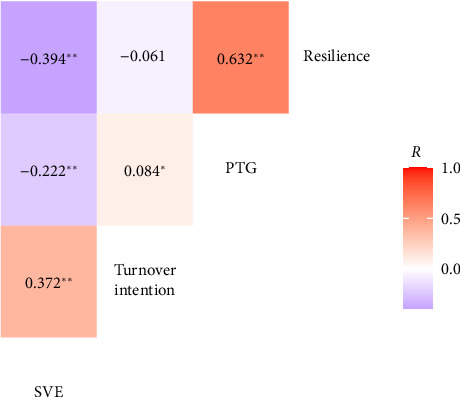
Correlations among SVE, turnover intention, PTG, and resilience. Note: ^∗^*p* < 0.05; ^∗∗^*p* < 0.01.

The total scores of the SV experience, resilience, PTG, and turnover intention among nurses were (64.86 ± 11.32), (26.52 ± 8.13), (62.12 ± 11.32), and (16.05 ± 4.42), respectively. Pearson's correlation analysis revealed that the SV experience was positively correlated with turnover intention (*r* = 0.372, *p* < 0.01), while negatively correlated with PTG (*r* = −0.222, *p* < 0.01) and resilience (*r* = −0.394, *p* < 0.01); PTG was positively correlated with resilience (*r* = 0.632, *p* < 0.01), while resilience was not significantly correlated with turnover intention (*r* = −0.061, *p* > 0.05). The specific correlations are shown in Figure 2.

### 3.3. Examination of Hypothesis

#### 3.3.1. Examination of Mediation Effect

To test the mediation effect of PTG between SV experience and turnover intention, we used the mediation model (Model 4) from the SPSS macro compiled by Hayes (2012). We employed a bias-corrected nonparametric percentile bootstrap method with 5000 resamples in line with the research hypothesis. The results indicated that the SV experience significantly positively predicted turnover intention (*β* = 0.161, *p* < 0.001) and significantly negatively predicted PTG (*β* = −0.381, *p* < 0.001); PTG significantly positively predicted turnover intention (*β* = 0.040, *p* < 0.001). After controlling for PTG, the value of the indirect effect was −0.015, and the 95% confidence interval for the indirect effect was [−0.027, −0.006], which does not overlap with zero, indicating a significant mediating effect of PTG between the SV experience and turnover intention. The indirect effect accounted for 9.3% of the direct effect. The indirect effect accounted for 9.3% of the direct effect (Table 2).

#### 3.3.2. Examination of the Moderated Mediation Effect

Model 8 from the SPSS macro compiled by Hayes (2012) was used to examine the moderated mediation effect in this study, assuming that both the first path of the mediation model and the direct path are moderated. This model aligns with the hypothesis of the present study. Results showed that, after incorporating resilience into the model, the interaction term between SV experience and resilience significantly predicted both PTG and turnover intention (turnover intention: *β* = 0.005, *t* = 2.851, *p* < 0.01; PTG: *β* = 0.016, *t* = 2.799, *p* < 0.01), this indicates that resilience moderated the direct effect of SV experience on turnover intention, as well as its effect on PTG. The detailed results are presented in Tables 3 and 4.

We categorized resilience into three groups, low, medium, and high resilience, to further explore its moderated effect. For nurses with low resilience (M − 1SD), the SV experience did not significantly predict PTG (*β* = −0.108, *t* = −1.293, *p* > 0.05). In contrast, for nurses with high resilience (M+1SD), the SV experience positively predicted PTG (*β* = −0.158, *t* = 2.235, *p* < 0.05), indicating that as nurses' resilience increases, the negative predictive effect of SV experience on PTG gradually diminishes, while the positive predictive effect gradually strengthens.

Similarly, for nurses with low resilience (M − 1SD), the SV experience significantly positively predicted turnover intention (*β* = 0.114, *t* = 5.080, *p* < 0.01). For nurses with high resilience (*M*+1SD), the SV experience also significantly positively predicted turnover intention (*β* = 0.188, *t* = 9.815, *p* < 0.01), indicating that as nurses' resilience increases, the predictive effect of SV experience on the turnover intention showed a gradually increasing trend.

## 4. Discussion

In the current study, we found that the SV experience of nurses in China was medium and had a significant mediating effect on turnover intention, and resilience moderated both the direct and mediating pathways in the model of SV experience, PTG, and turnover intention.

The level of SV experience in the current study was medium (64.86 ± 11.32), lower than that of previous research [12, 33, 34]; we posit that the higher levels of SV experience reported in those surveys can be primarily attributed to the fact that the participants in those studies were nurses working in ICU department, who are often exposed to higher levels of stress and trauma [4, 33]. Consistent with previous studies [3, 15, 19], we found a positive relationship between SV experience and turnover intention, meaning that the more severe distress experienced by nurses leads to a stronger turnover intention.

Despite rigorous quality control measures aimed at improving patient safety, adverse events remain inevitable due to the high complexity of healthcare systems. The SV phenomenon can increase the intention of healthcare personnel to resign, which raises nursing safety risks and leads to an increase in subsequent adverse events [35], seriously impacting the quality of nursing services.

Remarkably, the PTG partially mediated the relationship between the SV experience and turnover intention, indicating that the SV experience can directly affect the turnover intention and exert an indirect effect through PTG. Correlational analysis revealed a negative association between SV experience and PTG, indicating that the stronger the trauma-related distress and the weaker the perceived social support, the lower the PTG among nurses. Previous studies have noted that severe psychological suffering and perceived burden often predict poorer psychological outcomes [36], such as psychosomatic symptoms and occupational distress. Social support has been recognized as an important protective factor for PTG [37] and can foster PTG by creating meaning and facilitating positive reevaluations. Thus, after suffering adverse events, it is crucial for hospital administrators, colleagues, and family members to provide nurses with a safe environment, supportive atmosphere, and psychological backing. Such provisions can help mitigate negative emotions and promote adaptive outcomes.

Furthermore, our study highlights that PTG is positively correlated with turnover intention. PTG emphasizes individual growth and contributes to the reconstruction of self-perception and worldview. The occurrence of adverse nursing events triggers nurses' crisis awareness and self-reflection. On the one hand, this process may strengthen psychological resources, thereby mitigating emotional exhaustion and depersonalization while enhancing their ability to adapt to stress and challenges [20]. On the other hand, it may inspire individuals' willingness for change and proactive behavior, prompting them to engage more actively in career exploration, seek new opportunities, and pursue professional development, ultimately leading to career transition and growth [38]. Accordingly, once PTG reaches a certain threshold, it may motivate career change, manifesting as increased turnover intention. Overall, the mediating effect of PTG mitigates the negative impact of SV experience on turnover intention.

The current study found that resilience moderates the first half and direct path of SV experience ⟶ PTG ⟶ turnover intention mediation. This underscores the critical role of resilience for nurses when experiencing adverse events, affecting their PTG and turnover intention [39]. Specifically, as resilience increases, the negative predictive effect of SV experience on PTG gradually diminishes and may even turn positive. According to stress theory, stress can lead to positive growth if it remains below an individual's threshold, but it tends to result in stress injury when it exceeds that threshold. Thus, when nurses perceive a lower level of SV experience, a positive correlation with PTG emerges; conversely, a high level of perceived SV experience correlates negatively with PTG, resembling an inverted “U-shaped” relationship [13]. Our findings identified that nurses with low level of resilience were less likely to experience PTG and more likely to suffer from SV experience as they might have fewer psychological resources to cope with trauma. Nurses with higher resilience possess better psychological resources and dynamic regulatory abilities, resulting in a higher stress threshold and lower sensitivity to SV experience, allowing them to better manage the stress of SV experiences and achieve PTG and career success [40].

However, it is noteworthy that resilience enhances the direct effect of SV experience on turnover intention. Stress responses and outcomes are closely related to the intensity of trauma; within a certain range of trauma, a high level of resilience can indeed promote qualities such as resilience, strength, and optimism, which aids them in reconstructing emotional barriers and actively coping with trauma, thereby alleviating psychological distress associated with SV experience and reducing burnout [41]. Nevertheless, as the negative effects of SV experience intensity, nurses with high level of resilience may become more inclined to explore new possibilities and career opportunities, ultimately leading to an increase in turnover intention. Therefore, the moderating effect of resilience on the relationship between SV experience and turnover intention is a dynamic and complex process. According to our results, nursing managers should also consider the level of resilience of nurses in the intervention program aimed at reducing the SV experience and turnover intention and specify personalized support strategies according to different levels of resilience and PTG.

### 4.1. Implications for Nursing Management

Based on the current survey, hospital managers are recommended to consider comprehensively both the level of SV experience and resilience among nurses who suffered from adverse events Specifically, it is suggested to establish corresponding organizational support programs and create a positive hospital safety culture. These initiatives aim to safeguard nurses' mental health and reduce their intention to quit, thereby fostering a healthier work environment and retaining nursing talent. We suggest they financially invest in supporting programs for all nurses to protect themselves from adverse events. To ensure early detection of second victims, timely intervention, and regular evaluation, measures should be implemented to assist nurses affected by adverse events in recovering quickly [41]. This approach not only enhances their overall well-being but also contributes to the promotion of patient safety. According to the recovery process of the SV after adverse events [2], hospital support programs should provide psychological counseling, peer support groups, and debriefing sessions for nurses who experienced adverse events [43]. Meanwhile, creating a supportive environment of openness and support where nurses feel safe reporting adverse events without fear of blame or punishment and improving the patient safety culture would enhance the organizational support to decrease the SV experience [44]. Finally, early identification and intervention are still essential parts for improving nurses' mental health and decreasing turnover intention. Regular assessment of nurses' mental health, resilience, and turnover intention can help identify those at risk and provide targeted support, such as mindfulness training to improve occupational burnout [45]. All these strategies would be useful in implementing comprehensive measures to promote recovery based on individualized needs to reduce turnover intention.

### 4.2. Limitations

Given that the mediating effect was relatively modest, future research should aim to increase the sample size and consider incorporating additional relevant variables. Secondly, it would also be beneficial to stratify the SV experience based on the severity of trauma and the timing of its occurrence. Thirdly, although we included nurses from different hospitals in Zhejiang Province, the sample was limited to a single geographical area in China. Future studies ought to broaden data collection to encompass multiple centers across diverse regions and healthcare settings to enhance generalizability of the findings. This approach may provide deeper insights into how adverse events affect both positive and negative outcomes for nurses, thus offering new perspectives for developing and intervening support systems for SVs.

## 5. Conclusion

This study developed a moderated mediation model to examine the interrelationships among three key constructs: nurses' SV experience following adverse events, their positive outcomes (PTG), and negative outcomes (turnover intention). It analyzes and validates the mediating role of PTG and the moderating effect of resilience. Specifically, the evidence from the current study implies that the SV experience of nurses directly impacts the turnover intention, while the mediating effect of PTG mitigates the negative influence of the SV experience on this adverse outcome. Additionally, resilience moderates the impact of SV experience on both turnover intention and PTG.

## Figures and Tables

**Table 1:** Characteristics of participants in the study (*n* = 572).

Characteristics	Categories	*n* (%)
Gender	Female	537 (93.89)
	Male	35 (6.11)

Age	≤ 25	55 (9.62)
	26∼	137 (23.95)
	31∼	305 (53.32)
	≥ 41	75 (13.11)

Education	Under bachelor	59 (10.31)
	Bachelor	485 (84.79)
	Master	28 (4.90)

Years of working (year)	< 3	48 (8.39)
	3∼	68 (11.89)
	6∼	174 (30.42)
	11∼	221 (38.64)
	≥ 21	61 (10.66)

Professional qualification	Primary	263 (45.98)
	Intermediate	269 (47.03)
	Advanced	40 (6.99)

Whether have heard “second victim” before	Yes	173 (30.24)
	No	399 (69.76)

Department	Internal medicine	190 (33.22)
	Surgical	183 (31.99)
	Intensive or emergency	109 (19.06)
	Others (clinic, pediatric, etc.)	90 (15.73)

Type of adverse event	Unplanned extubation	106 (18.53)
	Medication	157 (27.45)
	Fall	190 (33.22)
	Suicide	38 (6.64)
	Pressure ulcers	81 (14.16)

*Note:* Professional qualification refers to the professional technical level of professional; typically, it can be clarified into primary, intermediate, and advanced.

**Table 2:** Direct and indirect effects and 95% confidence intervals for the model.

Effect	Estimated	SE	95% CI	
			Upper bound	Lower bound
Total effect	0.146	0.015	0.116	0.175
Direct effect	0.161	0.015	0.131	0.191
Indirect effect	−0.015	0.006	−0.027	−0.006

Abbreviation: CI = confidence interval.

**Table 3:** Moderated mediation model.

Independent variable	Dependent variable: PTG			Dependent variable: turnover intention		
	*B*	SE	*t*	*B*	SE	*t*
SVE	−0.409	0.176	−2.434^∗^	0.031	0.048	0.656
Resilience	0.438	0.402	1.089	−0.308	0.108	−2.847^∗∗^
PTG				0.039	0.011	3.429^∗∗∗^
SVE × resilience	0.016	0.006	2.799^∗∗^	0.005	0.002	2.851^∗∗^
*R* ^2^		0.408			0.179	
*F*		130.424^∗∗∗^			30.992^∗∗∗^	

Abbreviations: PTG = posttraumatic growth; SVE = second victim experience.

^∗^ *p* < 0.05.

^∗∗^ *p* < 0.01.

^∗∗∗^ *p* < 0.001.

**Table 4:** Direct and mediation effects on different levels of resilience.

	Resilience	Estimated	SE	95% CI	
				Lower bound	Upper bound
Direct effect	18.39 (M − 1SD)	0.114	0.023	0.070	0.158
	26.52 (M)	0.151	0.016	0.119	0.183
	34.65 (M + 1SD)	0.188	0.019	0.150	0.225

Mediation effect of PTG	18.39(M − 1SD)	−0.004	0.004	−0.012	0.001
	26.52 (M)	0.001	0.002	−0.004	0.006
	34.65 (M + 1SD)	0.006	0.004	0.000	0.015

## Data Availability

The data that support the findings of this study are available from the corresponding author upon reasonable request.
